# How Environmental and Ecological Stressors Reprogram Honey Bee Chemistry Through the Microbiome–Metabolome Axis

**DOI:** 10.3390/insects17030336

**Published:** 2026-03-19

**Authors:** Yahya Al Naggar, Hamed A. Ghramh, Amira Elfarnawany, Amr Mohamed

**Affiliations:** 1 Central Labs, King Khalid University and Center of Bee Research and its Products (CBRP), King Khalid University, AlQura’a, P.O. Box 960, Abha 61413, Saudi Arabia; halgramh@kku.edu.sa; 2 Zoology Department, Faculty of Science, Tanta University, Tanta 31527, Egypt; amira.elfarnawany@science.tanta.edu.eg; 3 Biology Department, Faculty of Science, King Khalid University, P.O. Box 9004, Abha 61413, Saudi Arabia; 4 Department of Entomology, Faculty of Science, Cairo University, Giza 12613, Egypt; mamr@sci.cu.edu.eg

**Keywords:** honey bees, gut microbiome, metabolomics, functional dysbiosis, environmental stressors, multi-omics

## Abstract

Honey bees are exposed to a wide range of environmental and ecological stressors that threaten individual health and colony sustainability. Growing evidence suggests that many of these stressors converge on a common target: the gut microbiome and its metabolic functions. The honey bee microbiome–metabolome axis represents a central regulatory system linking microbial symbionts with host nutrition, detoxification, immune competence, neural signaling, and social behavior. This review synthesizes current knowledge on how major stressors—including pesticides, antibiotics, pathogens, nutritional imbalance, thermal stress, habitat change, and environmental contaminants—reprogram honey bee chemistry by disrupting microbial community structure and, importantly, microbial and host metabolic pathways. We highlight recurring patterns consistent with functional dysbiosis, characterized by impaired energy metabolism, reduced production of short-chain fatty acids, altered amino acid and lipid metabolism, compromised antioxidant and detoxification capacity, and weakened immune regulation. However, much of the current evidence is correlative and derived from short-term or laboratory-focused studies; longitudinal and multi-site field validation of causal links remains limited. Importantly, emerging multi-omics studies suggest that profound metabolic disturbances can occur even when taxonomic changes in the microbiome are modest, emphasizing the need to move beyond descriptive community profiling toward functional and mechanistic assessments. We further discuss how stress-induced metabolic reprogramming at the individual level scales up to influence behavior, division of labor, and colony-level resilience. Finally, we propose a conceptual model illustrating how diverse stressors converge to disrupt the microbiome–metabolome axis, potentially leading to functional dysbiosis and host impairment.

## Simple Summary

Honey bee gut microbiota aids immune system function, detoxification, nutritional absorption, and digestion. Pesticides, antibiotics, diseases, inadequate nutrition, temperature stress, habitat change, and pollution all have an impact on honey bee metabolism and gut microbiome. This review demonstrates that a range of stressors frequently cause similar metabolic disorders, such as decreased energy generation, lower levels of beneficial microbial metabolites, impaired antioxidant and detoxification systems, and impaired immunity. Crucially, even minor changes in the composition of microorganisms can have a significant impact on metabolism. Focusing on how stressors affect microbial function, rather than only microbial composition, can improve our understanding of honey bee health decline and help develop better strategies for protection and conservation.

## 1. Introduction

Honey bees are essential pollinators that support wild-plant reproduction and the pollination of the majority of globally important crops (≈>70%) [1,2]. They are exposed to multiple interacting stressors—habitat loss, pesticides, climatic shifts, nutritional limitation, pathogens, *Varroa* infestation, and perturbation of the gut microbiome—which together undermine colony performance and contribute to elevated colony losses [3,4,5,6,7]. To link these environmental pressures to organismal and colony outcomes, it is increasingly useful to focus on the microbiome–metabolome axis: microbial symbionts both produce and modulate small-molecule metabolites (fermentation products, signaling compounds, and intermediates of host metabolism) that directly influence nutrition, detoxification, immune function, and behavior [8,9,10]. Consequently, metabolomics and integrated multi-omics approaches provide the functional readout necessary to move beyond taxonomic descriptions, enabling mechanistic connections between microbial change and physiological or ecological impact [11,12].

Metabolomics provides a systems-level view of the biochemical state of honey bees at a given time, integrating signals from nutrition, hormonal regulation, microbial interactions, xenobiotic detoxification, and chemical communication into a unified functional profile [13]. Evidence from metabolomic studies indicates that gut microbiota plays a central role in shaping host metabolic processes [14]. For instance, comparisons between microbiota-depleted bees and bees harboring their characteristic gut communities reveal pronounced differences in hemolymph and gut metabolite composition, including alterations in amino acids, vitamins, short-chain fatty acids (SCFAs), and other metabolites linked to nutrient assimilation and energy metabolism [15]. Moreover, individual microbial taxa can generate distinctive metabolic signatures within the gut environment, with some taxa specializing in carbohydrate- or lipid-derived metabolites, whereas others influence amino acid metabolism or modulate host hormonal signaling pathways [10].

Beyond the gut, metabolomics describes how the bees, including *Apis* and non-*Apis* species, break down chemicals from other sources, such as pesticide residues, as an example of plant protection products (PPPs) or secondary metabolites from plants derived from pollen. For instance, the chemical components of the food (real pollen vs. substitute feeds) dramatically change the internal metabolite landscape, affecting lipids, vitamins, phytochemicals, and stress-resilience markers [16]. Metabolomics has also been used to track how phytochemicals from diet affect pesticide accumulation or degradation, thus providing information about how floral resources and foraging conditions underlie colony health [17].

Finally, metabolomics serves as a bridge between individual physiological data and overall colony health data [13]. Chemical signals that underpin social regulation (e.g., pheromones influencing caste, reproduction, and foraging) eventually rely on metabolic pathways—from biosynthesis to breakdown, which may be tracked metabolically [18]. Crucially, metabolomics makes it possible to identify abnormalities in these pathways at an early stage, exposing physiological disruptions brought on by dietary modifications, environmental contaminants, pathogenic microbes, or imbalances in the microbiome long before obvious deteriorations in colony health or individual performance become noticeable [13].

**Figure 1:**
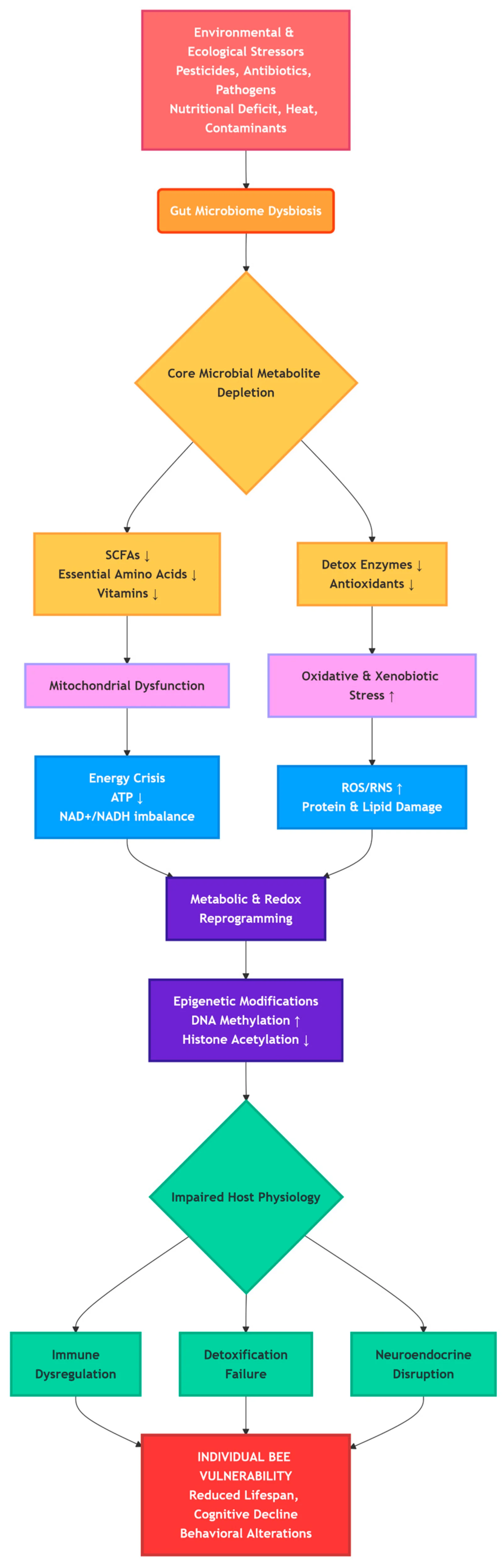
Conceptual model. Diverse environmental stressors drive gut dysbiosis, depleting core microbial metabolites (e.g., SCFAs) and reducing microbial detoxification capacity. This loss precipitates host metabolic collapse characterized by mitochondrial dysfunction and elevated oxidative stress, which alters cofactor availability for epigenetic enzymes and promotes DNA methylation and histone acetylation changes. Those epigenetic shifts can stabilize maladaptive states of immune dysregulation, impaired detoxification, and neuroendocrine disruption, ultimately determining individual bee resilience or vulnerability.

This review aimed to provide an integrative synthesis of how environmental and ecological stressors disrupt the honey bee microbiome–metabolome axis and, consequently, individual physiology and colony-level health. We examine the roles of gut symbionts in nutrition, detoxification, and immune regulation, and highlight how pesticides, antibiotics, pathogens, nutritional stress, temperature extremes, habitat changes, and environmental contaminants reprogram metabolic and functional pathways. Evidence on microbial transformations of dietary and xenobiotic compounds, metabolomic stress markers, functional dysbiosis, and the cascading effects of individual metabolic alterations on social behavior and colony resilience is presented. Finally, we discuss emerging multi-omics tools and intervention strategies that can advance understanding of stress-induced dysregulation and inform sustainable approaches to honey bee health management. To integrate these multifaceted interactions, we propose a conceptual model (Figure 1) that delineates the common pathways through which disparate environmental stressors reprogram host chemistry via microbial dysbiosis and metabolic disruption.

This review further advances existing syntheses by framing the microbiome–metabolome relationship as a functional, multi-scale axis rather than primarily a taxonomic description. We emphasize metabolic function as the key mediator linking microbial change to host bioenergetics, neural signaling, epigenetic cofactors, individual behavior, and emergent colony properties, while explicitly distinguishing experimentally supported mechanisms from associative or hypothesized links. In doing so, we provide an evidence-structured synthesis and a prioritized research roadmap to facilitate the transition from correlation-based observations toward causal, field-validated understanding.

This review integrates peer-reviewed research examining interactions between the honey bee microbiome and metabolome under environmental and anthropogenic stressors. A structured literature search was conducted using Web of Science, Scopus, PubMed, and Google Scholar for studies published between 2000 and January 2026, using combinations of keywords related to gut microbiota/microbiome, metabolomics/metabolome, pesticides, antibiotics, pathogens, diet, temperature stress, habitat change, and environmental contaminants in honey bees (*Apis mellifera*). Peer-reviewed articles written in English that applied microbiome profiling, metabolomics, or multi-omics approaches were considered. Titles and abstracts were screened for relevance, followed by full-text evaluation and cross-checking of reference lists to identify additional relevant studies. The selected literature was examined to identify recurring patterns, mechanisms, and knowledge gaps linking microbiome composition, metabolic function, and honey bee health at both individual and colony levels.

## 2. Historical Synthesis: The Evolution of Microbiome Research in Honey Bees

Honey bee microbiome research progressed rapidly from culture-based surveys to genome-aware ecology after the advent of high-throughput sequencing and reference genomes. Early culture work emphasized culturable bacteria in hive products and pathogen surveillance, while the release of the honey bee reference genome (2006) provided a host framework to relate microbial surveys to host biology [19]. A notable 2007 shotgun metagenomic study of colonies affected by Colony Collapse Disorder (CCD) reported shifts in viral and microbial signatures associated with collapsing hives; however, those early field-scale metagenomic results were associative and did not establish causal links between microbiome change and collapse [20]. Subsequent 16S and metagenomic surveys over the following decade clarified that adult worker hindguts are dominated by a small, conserved set of host-restricted phylotypes (e.g., *Snodgrassella*, *Gilliamella*, *Lactobacillales*) [21], and genome-resolved analyses later revealed extensive functional differentiation among those taxa [22].

Over the last decade, manipulative and gnotobiotic experiments have translated descriptive patterns into experimentally supported mechanisms in controlled settings. Laboratory studies show that perturbing the core gut community (for example, with antibiotics) can increase mortality and susceptibility to opportunistic infections in bees, and that microbial reconstitution can alter nutrient processing and weight trajectories under experimental conditions [15,23]. These interventions provide strong evidence for microbial contributions to digestion, immune modulation, and certain metabolic phenotypes within controlled systems, but caution is warranted when extrapolating these results to wild or managed colonies in complex field environments. At the same time, shotgun metagenomics and strain-level surveys have revealed deep genomic diversity within core phylotypes and documented geographic, seasonal, and agrochemical influences on community composition, motivating applied approaches (probiotics, microbial engineering) while underscoring persistent knowledge gaps—larval microbiome ontogeny, phage dynamics, and validation of causal microbiome–health links under multi-stressor field conditions [14,24]. Together, these lines of evidence make the honey bee a tractable model for mechanistic microbiome work, while also emphasizing the need for rigorous field validation.

## 3. Environmental and Ecological Stressors Disrupting the Microbiome–Metabolome Axis

The honey bee microbiota has a reduced, yet complex, microbiome, with important bacteria such as *Snodgrassella alvi*, *Gilliamella apicola*, *Lactobacillus Firm-4/Firm-5*, *Bifidobacterium*, and *Frischella perrara*, which are involved in nutritional metabolism, detoxification, immune protection, and the regulation of immune system homeostasis [14]. Environmental stress, caused by pesticides, antibiotic residues, pathogens, malnutrition, temperature fluctuations, and environmental contamination, acts as an inhibitor of microbiota or microbial metabolites. They could result in impaired detoxification functions and reduced production of SCFAs and immune system-related metabolites. These changes, in turn, may lead to metabolic disturbances [14], as explained for each type of environmental stress exposure in the following subsections.

### 3.1. Pesticides

Pesticides and inhive-chemicals represent some of the most widely reported and well-documented disruptors of the honey bee gut microbiota and related metabolic processes [25]. Table Pesticides can disrupt gut microbiota in two main ways: (i) by directly influencing the microbes’ growth, and (ii) by interfering with the host’s capacity to regulate its gut microbial community, such as by compromising host health or changing the gut environment [26]. The effects of pesticides on honey bee gut microbiota composition and metabolic functions are summarized in Table 1.

Neonicotinoids are a detrimental class of insecticides that can disrupt microbial communities in bees by reducing immunity through suppression of NF-kB/Relish signalling—an action that has been demonstrated to be microbiota-independent using a germ-free *Drosophila melanogaster* model [27]. Neonicotinoid-associated immunosuppression in both social (honey bees) and nonsocial (*D. melanogaster*) insects results in a loss of microbial control and increased vulnerability to infection by opportunistic pathobionts (e.g., *S. marcescens*) [26]. However, examining functional changes is crucial, as alterations in the structure of microbial communities do not invariably indicate modifications in community function.

For the first time, PICRUSt was used to compare the functional profiles of the bee gut microbiota before and after exposure for 6 weeks to the fungicide chlorothalonil (10 g/L) under hive conditions. The research team found that gene families linked to oxidative phosphorylation had higher anticipated gene counts, while gene families linked to sugar metabolism and protease activity had lower estimated gene counts [28]. Using LC-MS/MS analysis, the effects of three days of exposure to an environmentally relevant concentration (5 mg/L) of the fungicide carbendazim on the metabolic profile of honey bee workers were examined [29]. Bees treated with carbendazim had significantly different levels of 112 metabolites than the control group. High abundance and enrichment for a variety of pathways were observed in metabolites linked to energy and amino acid metabolism. Furthermore, the down-regulation of Aflatoxin B1exo-8,9-epoxide-GSH and glycerol diphosphate indicated that carbendazim may have an impact on honey bees’ immune systems and detoxification [29]. Similarly, the exposure of honey bee workers to a fungicide mixture named Chunmanchun^®^ (7% propiconazole and 28% carbendazim) at levels ranging from 0.159 g/L to 1.011 g/L for six days changed the activity of protective enzymes as well as the composition of the honey bee gut microbiota. *Lactobacillus* reduced by approximately 13%, while *Bartonella* and *Snodgrassella* rose by about 10% and 7.5%, respectively. Untargeted metabolomic analysis of hindgut samples using LC-MS/MS demonstrated that Chunmanchun^®^ treatment resulted in a quantitatively greater up-regulation of metabolites than down-regulation. The enriched pathways were mainly involved with lipid metabolism, signal transduction, neuronal function, immunity, the digestive system, amino acid metabolism, and the endocrine system [30].

Neonicotinoid pesticides, such as imidacloprid, thiamethoxam, or clothianidin, diminish the absolute abundance and functional role of dominating bacteria such as *Snodgrassella alvi* or *Gilliamella apicola*, which are critical for carbohydrate metabolism and biofilm development [31]. Such disturbances hinder the path of processing plant polysaccharides, as well as the subsequent synthesis of important compounds such as SCFAs, which are utilized by the gut epithelial cells for energy and for maintaining immune homeostasis [32]. The metabolic changes in the heads of honey bees exposed to 2 mg/L of the neonicotinoid pesticide thiacloprid for 3 days were studied using LC-MS and GC-MS metabolomic methods. The results showed that there were 115 metabolites significantly altered with high levels of abundance, enriched in a wide range of pathways associated with oxidative stress and detoxification [33]. Notably, adult and larval worker honey bees exposed to sucrose solutions containing acetamiprid neonicotinoid insecticide at concentrations of 0, 5, and 25 mg/L for seven days (adults) and four days (larvae) showed a significant increase in the relative abundance of *Commensalibacter*, while *Bifidobacterium* and *Gilliamella* levels decreased in adults, and *Bombella* significantly decreased in larvae. The reduction in *Bombella* was found to be significantly correlated with 27 out of 42 differential metabolites, including amino acid metabolism (55.56%, predominant), lipid metabolism, carbohydrate metabolism, signalling molecules and interactions, the biosynthesis of other secondary metabolites, and nucleotide metabolism [34].

Exposing honey bees to a field-relevant concentration of flumethrin acaricide (10 μg/L) for 14 days increased immunological responses (defensin upregulation) in the gut. Still, it had little impact on survival and gut microbial composition. However, untargeted metabolomics analysis using ultra-high-performance liquid chromatography-quadrupole time-of-flight mass spectrometry (UPLC-Q-TOF/MS) showed that flumethrin significantly altered the composition of intestinal metabolites. This metabolic stress was closely linked with a reduction in gut core bacterial endosymbiont *Gilliamella* spp., resulting in significant changes in glycerophospholipid metabolism in both the host and the bacteria [35].

Notably, the synergistic effects of pesticides enhance microbiome and metabolome disorders. For example, exposure of honey bee workers to field-relevant concentrations of flupyradifurone (0.0043 μg μL^−1^) or sulfoxaflor (0.000047 μg μL^−1^) insecticide with or without the fungicide azoxystrobin (0.038 μg μL^−1^) for 10 days, altered both bacterial and fungal communities of the gut microbiota, reduced beneficial microbial diversity and core microbial communities, concurrent with the enhancement of opportunistic pathogens such as *Serratia marcescens* [36]. The gut microbial network becomes unstable as a result of these cross-kingdom disturbances, endangering both the resilience and general health of bees [36,37]. These multi-chemical exposures, which are typical in agricultural environments, pose a serious risk to the long-term health and resilience of bee colonies. As a result, a unique methodology for improving risk assessment that considers the possible impacts of agrochemicals on the gut microbiota and metabolome of bees is urgently required.

**Table 1:** Reported effects of pesticides on honey bee gut microbiota composition and metabolic functions.

Pesticide/Chemical	Class	Exposure Conditions	Dose/Duration	Main Effects on Gut Microbiota and Metabolism	Reference
Chlorothalonil	Fungicide	Hive exposure	10 g/L for 6 weeks	Increased predicted gene families associated with oxidative phosphorylation; decreased gene families related to sugar metabolism and protease activity	[28]
Carbendazim	Fungicide	Oral exposure	5 mg/L for 3 days	Significant changes in 112 metabolites; enrichment in energy and amino acid metabolism pathways; down-regulation of detoxification-related metabolites	[29]
Propiconazole + Carbendazim (Chunmanchun^®^)	Fungicide mixture	Oral exposure	0.159–1.011 g/L for 6 days	*Lactobacillus* decreased (~13%); *Bartonella* increased (~10%); *Snodgrassella* increased (~7.5%); metabolomic alterations affecting lipid metabolism, immunity, and amino acid metabolism	[30]
Imidacloprid/Thiamethoxam/Clothianidin	Neonicotinoid insecticides	Oral exposure	Sublethal concentrations	Reduced abundance of core bacteria such as *Snodgrassella alvi* and *Gilliamella apicola*, affecting carbohydrate metabolism and SCFA production	[31,32]
Thiacloprid	Neonicotinoid insecticide	Oral exposure	2 mg/L for 3 days	115 metabolites significantly altered; enrichment in oxidative stress and detoxification pathways	[33]
Acetamiprid	Neonicotinoid insecticide	Oral exposure (adults and larvae)	0, 5, and 25 mg/L for 7 days (adults)/4 days (larvae)	Increased *Commensalibacter*; decreased Bifidobacterium and *Gilliamella* in adults; decreased *Bombella* in larvae; associated with metabolic changes in amino acid, lipid, and carbohydrate pathways	[34]
Flumethrin	Acaricide	Oral exposure	10 μg/L for 14 days	Minimal effect on microbial composition but reduction in *Gilliamella* spp.; significant changes in intestinal metabolites and glycerophospholipid metabolism	[35]
Flupyradifurone/Sulfoxaflor ± Azoxystrobin	Insecticides ± fungicide	Oral exposure	0.0043 μg μL^−1^ (flupyradifurone), 0.000047 μg μL^−1^ (sulfoxaflor), 0.038 μg μL^−1^ (azoxystrobin) for 10 days	Disruption of bacterial and fungal gut communities; reduced microbial diversity; increase in opportunistic pathogens such as *Serratia marcescens*; Disruption of the fungal and bacterial communities’ network and coexistence.	[36,37]

### 3.2. Antibiotics

In apiculture, antibiotics such as tetracycline, oxytetracycline (OTC), tylosin, and fumagillin have been commonly used prophylactically in several countries, but they can have severe and long-lasting detrimental effects on bee health [23,38]. These antibiotics significantly affect beneficial gut symbionts, including *Bifidobacterium*, *Lactobacillus* Firm-5, *Lactobacillus* Firm-4, and *Snodgrassella alvi*, which play essential roles in maintaining gut epithelial integrity, protecting against pathogens, and supporting immune system development [23].

Tetracycline treatment (450 µg/mL) demonstrated enduring effects on the size and composition of the honey bee gut microbiome, leading to reduced survivorship in both hive and laboratory settings when bees were exposed to opportunistic bacterial infections (*Serratia kz11*) [23]. Additionally, the gut microbiota of honey bee workers treated with tetracycline did not recover after being reintroduced to their colonies, indicating a high probability of a cascade effect of the treatment on their nestmates through social transmission of microbiota (fecal-oral transmission, oral-oral transmission, and contact with the hive components (comb, honey, and beebread) [23]. A follow-up study was carried out to validate the latter hypothesis using both hive and laboratory settings, and it revealed a significant change in beta diversity of the microbial community of workers colonised by a tetracycline-disrupted gut community compared to those colonised with a normal gut community, which may negatively affect the function of gut microbiota and, consequently, the physiology of their host [39]. A recent study also found that exposing honey bees to 450 μg/mL of tetracycline for five days in hive (nucs) environments altered the gut microbiome structure [40]. Thirteen of the thirty-three bacterial sub-lineages underwent substantial frequency changes, including a drop of 5/11 of *Bifidobacterium*, a rise of 4/7 of *Gilliamella*, a decrease of 2/2 of *Lactobacillus kunkeei*, and a decrease of 2/6 of *Lactobacillus Firm-5*. The delayed shift to foraging behaviour was found to be related to this microbial perturbation. Antibiotic exposure also decreased gut bacterial metabolic gene repertoire and brain neurotransmitter titers, most of which were related to metabolism, including energy, carbohydrates, amino acids, cofactors and vitamins, nucleotides, and lipids [40].

The effects of administering oxytetracycline (OTC) (135 μg/mL) to honey bee workers throughout their larval and/or adult stages under laboratory conditions were also recently examined [41]. A long-term survival crisis, weight loss, and decreased expression of immunological and developmental genes were observed in adult bees exposed to OTC. Importantly, larval antibiotic exposure affects the development of the gut microbiota in adult bees, and dual larval-adult exposure results in a synergistic dysbiosis of the gut microbiota. Furthermore, it has long-term effects on adult bees’ intestinal metabolism, albeit not as much as more recent exposure during the adult stage. In addition, the alterations in metabolism due to larval and adult treatment were less severe and more comparable to those in the larval treatment than those in the adult treatment alone. The alterations were due to a slight impairment of carbohydrate metabolism, changes in amino acid metabolism, a slight impairment of energy metabolism, changes in vitamin and cofactor metabolism, and reduced activity of nucleotide metabolism. Also, the detection of colonisation by *Paenibacillus* and subsequently by *Melissococcus* in either the larval or adult treatment group may hint at the fact that the community defence mechanism created by the host and its microorganisms against colonisation by pathogens is hampered by larval exposure to antibiotics, which bears semblance to its severity in larval developmental life [41,42,43].

### 3.3. Pathogens

Pathogens affecting honey bees encompass bacteria (American Foulbrood, European Foulbrood), fungi (Chalkbrood), viruses (Deformed Wing Virus (DWV), Sacbrood Virus (SBV)), and microsporidia (*Nosema* species), which are often disseminated by pests such as *Varroa* mites or via contaminated food or water, leading to colony deterioration, wing deformities, dysentery, and unpleasant odours [44]. These pathogens can disrupt the vital role of microbes in the microbiome–metabolome by interacting in multiple ways that directly alter metabolic processes.

*Nosema ceranae* is a gut pathogen that seriously harms honey bees by degrading their midguts, impairing nutrient assimilation, shortening lifespan, and reducing immunological capabilities [45,46,47]. These effects lead to declines in colony health, poor brood development, earlier foraging activity, reduced honey production, and, potentially, colony death [48]. Moreover, *N. ceranae* infection has been associated with changes in the bee gut microbiota, including increased abundance of *Proteus mirabilis*, *Frischella perrara*, and *Gilliamella apicola*, and decreased abundance of *Bifidobacterium asteroides*, *Fructobacillus fructosus*, and *Lactobacillus* spp., which may contribute to reduced honey bee survival. [49]. This gut parasite attacks the midgut epithelium, disrupts the microenvironment for symbiont establishment, and directly depletes host energy metabolites, such as carbohydrates and amino acids [38,50]. Notably, seasonality and bee age had a substantial impact on the bacterial structure and composition of the bee gut [51]. In the same sense, the age of the bees at the time of *N. ceranae* infection has an impact on the gut bacterial community. Elevated loads of *G. apicola*, *Bifidobacterium asteroides*, *Bombilactobacillus* spp., *Lactobacillus* spp., *Bartonella apis*, and *Bombella apis* were found in the infected bees shortly after emergence. Additionally, older non-infected bees had larger loads of *Bifidobacterium asteroides*, *Bombilactobacillus* spp., *Lactobacillus* spp., *Ba. apis*, and *Bo. apis*, while infected bees at almost all ages had higher loads of *G. apicola* [52].

Chalkbrood disease affects honey bee larvae worldwide and is caused by the fungus *Ascosphaera apis*, an obligatory fungal infection of honey bee brood. LC-MS-based untargeted metabolomics was used to examine the alterations in the metabolomic profiles in the gut tissues of *A. apis*-infected larvae (10^5^ *A. apis* spores per larva) and controls [53]. The guts of *A. apis*-infected larvae had levels of 28 and 52 metabolites that were considerably higher and lower, respectively, than those of control larvae, according to the metabolomic data. The amount of 5-oxo-ETE was twice as high in the infected larvae as in the control larvae, suggesting that it could be used as a metabolic biomarker to diagnose chalkbrood disease. The gut of *A. apis*-infected larvae had considerably lower levels of metabolites that fight oxidative stress, including taurine, docosahexaenoic acid, and L-carnitine. This suggests that *A. apis* infection may impair the larvae’s capacity to deal with oxidative stress [53].

The presence of a functional microbiota has been associated with higher survival rates and increased resistance of honey bees to DWV infection, suggesting a potential protective role of gut microbes [54]. Perturbation or reduction in the gut microbiota has been linked to increased host mortality when bees are challenged with the opportunistic pathogen *S. marcescens*. For example, *S. marcescensis* is quickly destroyed in the presence of microbiota but survives in the absence of microbiota in the bee gut. Protection is diminished in monocolonized and antibiotic-treated bees, probably because different symbionts occupy different niches [55]. Overall, shifts in microbiota composition following fungal or bacterial infection may impair the breakdown of complex carbohydrates and fermentation products inside the gut of bees, influencing the metabolic pathway due to disrupted nutrient distribution in the gut, resulting in higher susceptibility to secondary infections [14].

### 3.4. Nutritional Stress

Nutrition represents the main thrust of honey bee biology, and the variability of pollen diets enhances immunity and resistance to pathogens [56,57]. However, environmental or agricultural changes limit bees’ access to a variety of high-quality pollen and nectar sources, leading to nutritional stress and possibly increasing vulnerability to viral and fungal infections [58]. At the colony level, landscape diversity and the availability of floral resources strongly influence dietary diversity, with simplified agricultural landscapes and monoculture systems often providing limited and nutritionally imbalanced forage. Given the importance of the gut microbiome in metabolism, dietary variations/stress can directly affect the microbiota structure and its functional roles [59].

The diversity and quality of pollen affect microbiota stability and vulnerability to pathogens [60]. Feeding worker bees with aged pollen causes dysbiosis, characterized by reduced levels of *Snodgrassella alvi* and a corresponding increase in opportunistic non-core microbiota *Frischella perrara* and *Bombella apis*. In addition, the establishment of the common midgut pathogen *Nosema* spp. was substantially related to ileum dysbiosis and host inadequacies. Furthermore, dysbiosis in the ileum was replicated in the rectum, mouthparts, and hypopharyngeal glands, indicating a systemic host response [60]. Pollen shortage also alters microbiome composition and metabolic function. For instance, pollen-deprived bees had significantly lower abundances of key taxa such as *Lactobacillus*, *Bombilactobacillus*, *Bifidobacterium*, *Gilliamella*, and *Snodgrassella*. Bacterial fermentative enzyme gene transcripts (acetate kinase, lactate dehydrogenase, and hydroxybutyryl-CoA dehydrogenase) were expressed at lower levels in correlation with diet-induced microbiota changes [61]. Pollen diversity also affects microbiota, with bees fed monofloral diets having lower levels of *Lactobacillus*, *Bombilactobacillus*, and *Bifidobacterium* but higher levels of *Bartonella apis* and higher rates of *Nosema* infections compared to bees fed polyfloral diets [59,62].

The pollen-free diet significantly lowered the expression of genes (Vitellogenin (*Vg*) and Juvenile Hormone Esterase (*JHE*) that are essential for honey bee development, and these changes in gene expression may be linked to gut microbiota colonisation [63]. Furthermore, in the gut of bees with a defined bacterial population, transcript levels of the insulin receptor genes *AmInR1* and *AmInR2* were found to be lower than in microbiota-deficient bees. Furthermore, bees injected with a particular gut microbiota and grown on an artificial diet had a lower ability to control infection from a bacterial pathogen (*S. marcescens* KZ11) than those fed natural pollen [63]. Similarly, the gut microbiome of microbially depleted Africanized honey bees (*Apis mellifera scutellata* Lepeletier) raised in semi-sterile conditions and fed different protein diets was compared to wild honey bees collected during the flowering season [64]. Microbially depleted bees had more species richness and variety in their guts. However, in both wild bees and those fed the various protein diets, the non-core gut microbial community predominated. Important enzymes like β-glucosidase, β-galactosidase, pyruvate dehydrogenase, and phosphoglycerate mutase, which are essential for improving nutrition absorption, digestion, and carbohydrate metabolism, were also identified by functional predictions of the gut microbial population [64]. More research is still needed to explore the impact of dietary stress in combination with other ecological and environmental stresses on gut microbiota and metabolic profiles of bees, as well as the long-term consequences on bee health at the individual and colony levels.

### 3.5. Heat Stress

One of the most important environmental stressors affecting both human and animal health is heat stress, which mainly causes intestinal damage and dysbiosis in the gut microbiota [65,66]. In addition, plant microbial communities are also significantly altered by thermal stress [67]. Evidence from other insect models also suggests that temperature can strongly influence gut microbiota composition. For example, *Drosophila melanogaster* individuals reared at 31 °C exhibited higher levels of Acetobacter, the predominant Proteobacteria species, compared with those reared at cooler temperatures of 13 °C [68]. Similarly, in the terrestrial isopod *Porcellio scaber*, the abundance of antibiotic-producing Actinobacteria decreases with rising temperature, negatively affecting host growth [69].

Temperature also influences the interaction of honey bee gut symbionts and gut parasites. Laboratory studies reveal that temperature fluctuations impact microbiome composition and infection severity, boosting vulnerability to parasites in bumblebees [70,71]. Furthermore, a heat-resistant *Lactobacillus* symbiont is more successful in preventing the growth of a trypanosomatid gut parasite in honey bees through the production of organic acids (e.g., lactic acid and acetic acid) as metabolic byproducts, lowering the pH of the environment. Importantly, high temperatures increased this acid-mediated suppression because the bacterial symbiont grows and generates acids faster at warmer temperatures relative to the parasite [72]. It can therefore be noted that normal temperatures found in a bee colony can increase symbiont-linked efficacies for eliminating intestinal parasites.

Studies in other bee species, particularly bumblebees, have also examined the effects of temperature stress and pathogenic infection on gut microbial communities. While the core gut microbiome persisted, with species such as *Bombilactobacillus* and *Snodgrassila* unaffected by treatment, there were significant changes in other essential organisms. *Asaia bogorensis* (acetic acid bacteria) increased in control temperature-infected organisms, but lactic acid bacteria (*Lactobacillus* species) were surpassed at high temperatures by large increases in *Apilactobacillus kunkeei*. *Citrobacter freundii*, an opportunistic bacterial pathogen, prevailed in hot-infected bees, indicating an elevated immunocompromised status due to the combined stresses’ impact on bee gut health [73]. More research is needed to determine the effects of heat stress alone or in combination with other stressors on the bee gut metabolome. These findings from model insects and related bee species may help inform our understanding of how honey bees could respond to climate change.

### 3.6. Habitat Change

One of the main causes of pollinator decrease globally is thought to be habitat loss and nesting habitat degradation brought on by urban and agricultural expansion [74]. Natural habitats are replaced by increasing urbanisation, and the majority of the original ecosystems are lost locally, leading to local loss of original ecosystems, sharp declines in biodiversity, and, in some cases, local extinction of native species [75]. Habitat loss/change can impact the intestinal microbiota of insects, including bees, with potential consequences for health, including reduced immunity, higher susceptibility to infections, and impaired nutrient absorption [76].

However, studies explicitly linking landscape change to honey bee gut microbiota remain limited. Available evidence suggests that microbial assemblages are largely geographically conserved [77,78,79], but the extent to which habitat modification alters functional microbial profiles remains poorly understood. For example, comparative studies of the bacterial fraction of the apibiome in hives across agricultural, semi-natural, and natural environments revealed decreased community evenness and depletion of beneficial bacteria in agricultural settings. Arsenophonus abundance declined from agricultural to natural sites, potentially serving as a bioindicator of anthropogenic disturbance, and has been associated with increased bee mortality and reduced colony health [80,81,82]. Natural environments supported metabolic pathways, including pyrimidine and UMP synthesis, that were reduced in agricultural environments, suggesting that habitat change may compromise microbial-driven metabolic functions [78,81].

Similarly, urban–rural studies of small carpenter bees in Toronto indicated that pollen sources host higher microbial diversity than bees themselves. Urban environments maintain complex plant–microbe networks and higher microbial diversity, yet show lower relative abundances of beneficial symbionts and higher pathogen loads (e.g., *Ascosphaera*, *Alternaria*), whereas rural pollen contained more pesticide residues that could disrupt symbioses [82].

These findings indicate that habitat changes, driven by human activity, can influence microbial communities critical to bee health. Nevertheless, the literature remains limited, particularly regarding how specific habitat features (e.g., urbanization intensity, agricultural practices, plant diversity) affect microbial taxa and functional pathways over time. Further research is urgently needed to identify which taxa are enriched or depleted under different environmental pressures, the metabolic consequences for host insects, and the long-term impacts on colony resilience and ecosystem services.

### 3.7. Environmental Contaminants

Contaminants in the environment, for instance, heavy metals and microplastics (MPs), may interfere with the gut microbiota and metabolites of honey bees, which can negatively impact the host’s physiological condition. Honey bees exposed to sub-lethal levels of heavy metals, including cadmium or selenate, exhibit altered metabolic fingerprints and subtle changes in core microbiota. Specifically, metabolites involved in detoxification (e.g., glutathione [GSH], oxidized glutathione [GSSG], γ-Glutamyl amino acids, methionine), proteolysis (e.g., free amino acids such as leucine, isoleucine, valine, alanine, glycine; dipeptides and oligopeptides; urea; ammonia; putrescine; spermidine), and lipolysis (e.g., free fatty acids such as palmitic, stearic, oleic acids; acylcarnitines; glycerol; monoacylglycerols; lysophospholipids [LPCs, LPEs]; malondialdehyde-related lipid peroxidation markers) are altered, indicating impairment of gut metabolism and host responses to toxic stress [83].

MP exposure decreases gut microbial alpha diversity, alters the composition of core bacterial communities, and affects gene expression associated with antioxidative defense, immunity, and detoxification [84]. Polystyrene MPs, either alone or in combination with the insecticide flupyradifurone (FPF), increase oxidative stress (via catalase inhibition), impair detoxification (downregulation of CYP9q2), and reduce immunological function (downregulation of hymenoptaecin). Co-exposure also drastically reduces Lactobacillus abundance and alters gut microbiota composition [85]. Together, these studies highlight the susceptibility of the bee gut microbial ecosystem to environmental pollutants and demonstrate the broad metabolic and functional consequences of contaminant exposure.

## 4. Shared Effects of Diverse Stressors on the Gut Microbiota–Metabolome–Host Axis

As summarized in Figure 1, although stressors of both environmental and anthropogenic origins have diverse causes, their effects on the gut microbiota-metabolome-host system are remarkably similar. Dysbiosis is a persistent consequence of diverse stressors, defined by reduced instability of the microbial community, reduced levels of beneficial core microbes, and an increase in opportunistic/pathogenic microorganisms. Reduced levels of beneficial microbes refer, of course, to beneficial fermenters, as opposed to opportunistic pathogens, which include pathogens of the opportunistic sort.

**Figure 2:**
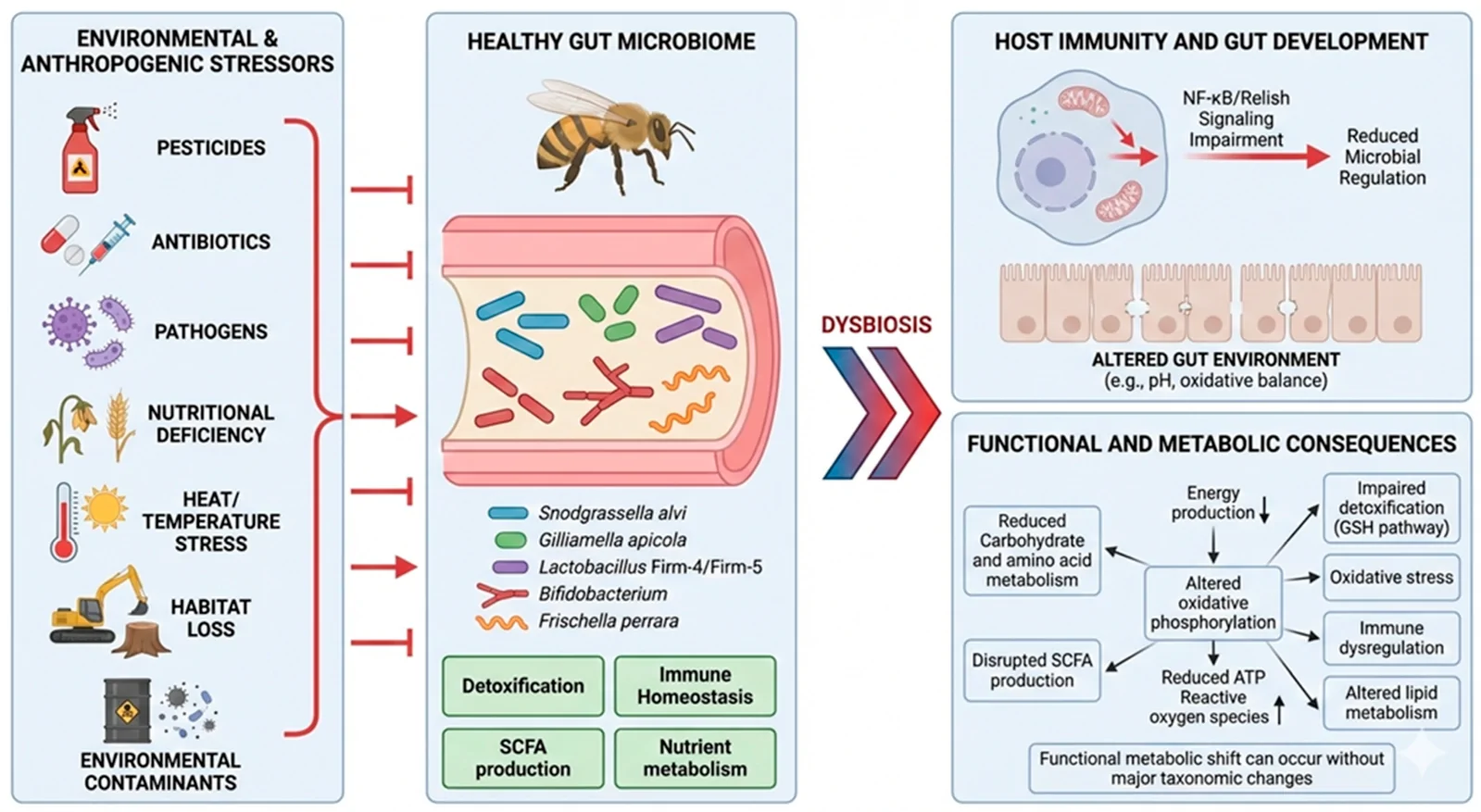
Exposure to stressors causes dysbiosis, an immune dysfunction due to impaired immune signaling, difficulties in gut morphogenesis, and changes in gut environments. These factors ultimately result in lowered energy function, impaired detoxification function, abnormal SCFA metabolism, oxidative damage, immune disruption, and an impaired immune system.

At the metabolic level, all stressors bring about disturbances in energy metabolism, such as disturbances in glucose fermentation, reduction in the production of SCFAs, disturbances in amino acid and lipid metabolism, and impaired oxidative phosphorylation, which often results in ATP reduction. Further, detoxification mechanisms, such as glutathione-related mechanisms and antioxidant mechanisms, are also often impaired, which leads to an augmentation in oxidative stress. One of the common features of dysregulation of the immune system is displayed by the immune responses of the host, which can be characterized by poor control of pathogens, impaired immune signaling, and an exaggerated susceptibility to secondary infection. Most importantly, dysregulation in metabolic and functional aspects can be exhibited independent of changes in the taxonomy of the microbe, proposing that functional dysbiosis can be one of the common features of stress (Figure 2, Table S1).

### Molecular Mechanisms of Stress-Induced Metabolic Reprogramming: Mitochondria and Epigenetics

Because direct experimental evidence linking these pathways in honey bees remains limited, the mechanisms discussed below should be interpreted as hypothesis-generating models informed by emerging bee studies and by mechanistic insights from other animal systems.

Beyond descriptive shifts in metabolite pools, recent studies have begun to identify molecular pathways through which stress-associated microbiome changes can influence host metabolism. One mechanistic node receiving increasing attention is mitochondrial function. Gut microbiota contribute metabolites, including short-chain fatty acids (SCFAs) such as butyrate, that are known regulators of mitochondrial biogenesis and cellular energy metabolism in many animal systems [14]. In honey bees, stressors including xenobiotics and microbiome disruption are associated with altered metabolite availability and signatures consistent with mitochondrial impairment, including reduced ATP production, elevated reactive oxygen species, and altered cellular redox balance (NAD^+^/NADH) [86]. Although direct causal pathways linking microbiome-derived metabolites to mitochondrial dysfunction in bees remain incompletely resolved, these observations are consistent with a role for microbial metabolic products in maintaining host bioenergetic stability.

**Figure 3:**
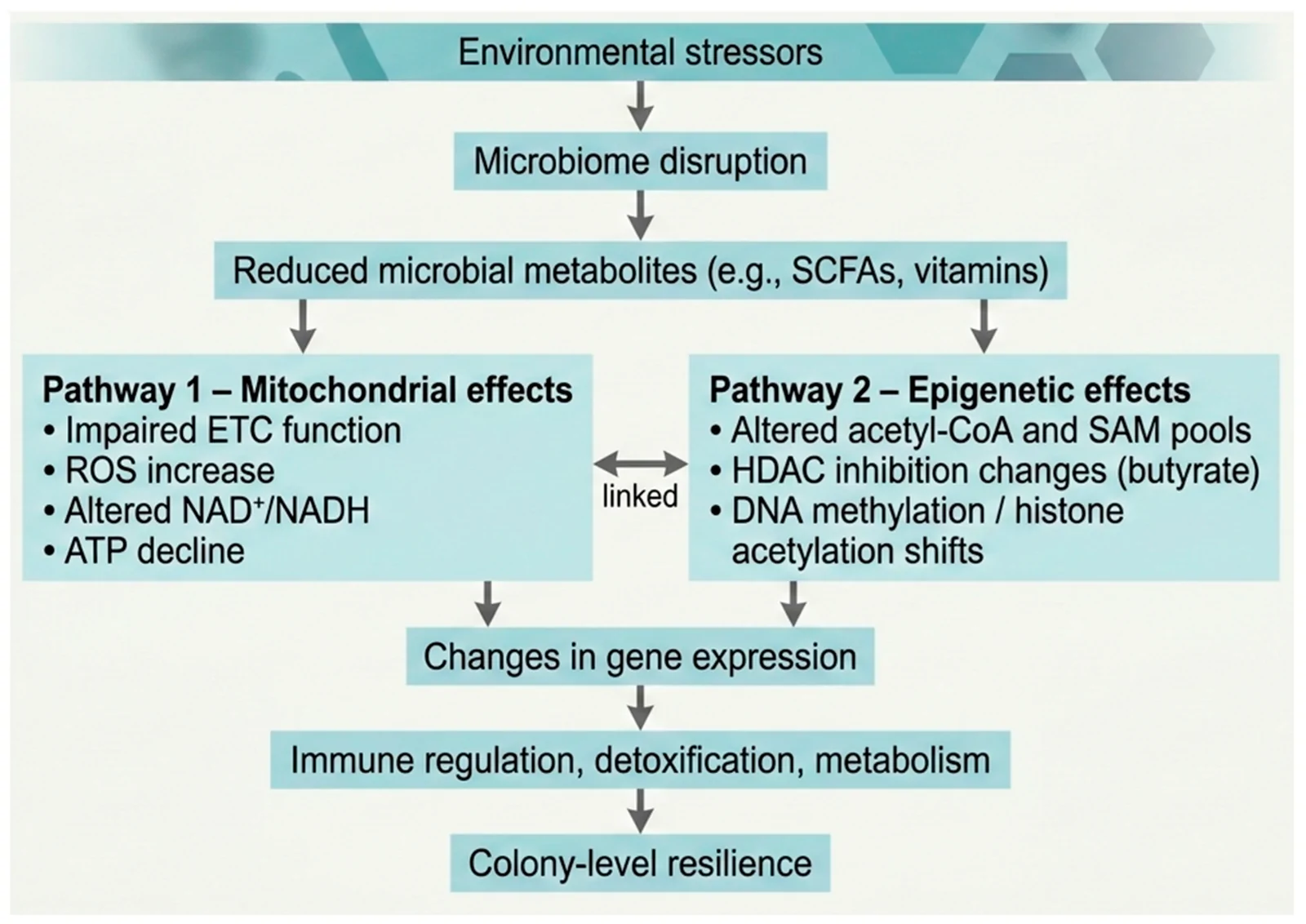
Proposed mechanistic links between microbiome metabolites, mitochondrial function, and epigenetic regulation in honey bees.

Metabolic and redox perturbations are also closely connected to epigenetic regulation. Many epigenetic enzymes depend on metabolic cofactors such as S-adenosylmethionine (for DNA and histone methylation) or acetyl-CoA (for histone acetylation), thereby linking cellular metabolic state to chromatin regulation [87]. In honey bees, pesticide exposure has been associated with altered DNA methylation patterns in genes related to immunity and detoxification pathways [88]. In addition, the microbiota-derived metabolite butyrate functions as a histone deacetylase inhibitor in diverse biological systems; reductions in butyrate during microbiome disruption may therefore influence host transcriptional regulation, although this mechanism remains to be experimentally validated in bees [89]. Taken together, these observations support a conceptual framework in which stress-induced microbiome perturbations alter microbial metabolite production, potentially influencing mitochondrial bioenergetics and the availability of epigenetic cofactors. While several components of this pathway have been individually documented in honey bees, the integrated causal relationships proposed here remain to be experimentally tested. These interactions may therefore contribute to persistent physiological effects following stress exposure, but targeted mechanistic and longitudinal field studies will be required to confirm these links in honey bee systems [90]. A schematic overview of these proposed interactions is presented in Figure 3.

## 5. Colony-Level Health: From Individual Metabolomes to Social Physiology

**Figure 4:**
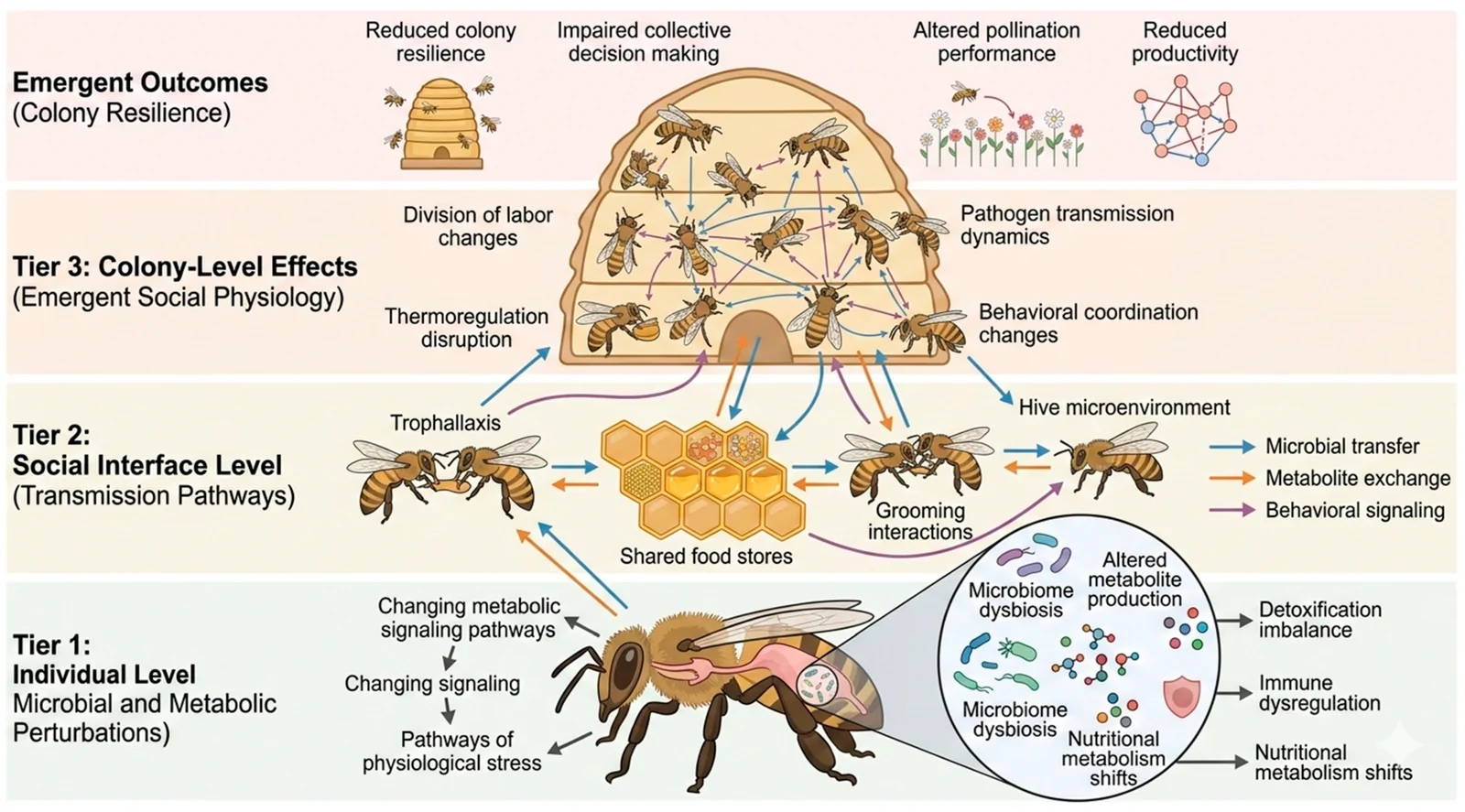
Multi-scale impacts of the microbiome-metabolome axis on colony social physiology. This diagram visualizes how individual-level microbial and metabolic disturbances propagate through social interfaces to affect colony-level resilience and function. Microbially driven changes in individual metabolism and signaling are transmitted via trophallaxis, shared food stores, grooming interactions, and the hive microenvironment, producing collective shifts in behavior, division of labor, thermoregulation, and pathogen dynamics. These propagated effects generate emergent colony properties, for example, altered social network robustness, impaired collective decision making, and reduced pollination performance, that ultimately define colony-level resilience and operational outcomes.

The gut microbiota may influence colony social complexity by shaping individual physiology and behavior (Figure 4, Table 2). Recent automated tracking studies report that workers with a conventional gut microbiota show greater head-to-head contact, stronger task specialization, and more persistent social associations than microbiota-depleted workers [91]. These behavioral differences are associated with changes in brain metabolites (for example, serine and ornithine) and altered expression of genes implicated in synaptic function and social responsiveness [91,92]. We emphasize that these studies are primarily associative: manipulative evidence directly linking specific microbiome shifts to altered colony-level behavior in realistic field conditions remains limited, and alternative explanations (for example, differences in nutrition, pathogen load, or rearing environment) cannot be fully excluded without targeted field manipulations.

Metabolomic and neuroanatomical evidence indicates crosstalk between gut microbial activity and amino-acid–linked pathways in brain regions associated with olfactory and gustatory processing (antennal lobes, subesophageal ganglion), which plausibly mediates changes in nestmate recognition and stimulus-driven behavior [93,94]. Multi-omics work comparing apiaries in contrasting landscapes has further shown large seasonal and site-dependent shifts in hemolymph protein and small-molecule profiles, including declines in vitellogenin and apolipophorin under intensive agricultural exposure [95]. These findings suggest potential routes by which environment-mediated alterations in microbial metabolism could scale to colony physiology, but we caution that direct causal demonstration at the field scale is still an active area for future research.

**Table 2:** Outlines how the honey bee microbiome shapes colony chemical communication, governing social organization. It links environmental stressor–induced microbiome disruption to failures in semiochemical signaling, providing a mechanistic framework for how individual dysbiosis can scale into colony-level dysfunction.

Semiochemical Class	Compound/Signal	Microbial Source/Modulator	Bee Life Stage/Caste	Behavioral/Social Role	Proposed Mechanism of Microbial Influence	Stressor Link & Reference
Contact Pheromones & Recognition Cues	Cuticular Hydrocarbons (CHCs)	Gut microbiota (e.g., *Snodgrassella alvi*, *Gilliamella apicola*, *Lactobacillus* spp.)	Workers, Queen	Nestmate recognition, task thresholds, and social cohesion	Microbiota-derived metabolites (SCFAs, amino acids) influence host lipid metabolism and CHC biosynthesis. Dysbiosis alters CHC profiles, potentially disrupting recognition.	Pesticides & antibiotics deplete key taxa, possibly altering CHC blends and leading to social rejection or impaired task coordination [10,14]
Queen Pheromones	Queen Mandibular Pheromone (QMP) components	Indirect modulation via gut microbiome-influenced host nutrition & metabolism	Queen	Inhibition of worker ovary development, retinue attraction, and colony cohesion	The microbiome supports the queen’s nutrition (vitamins, lipid metabolism), which is essential for pheromone synthesis. Dysbiosis may reduce pheromone titre or alter blend.	Nutritional stress & pathogens compromise queen health and pheromone output; microbiome disruption may amplify this [18].
Brood Pheromones	Brood ester pheromone	Potential modulation by hive & brood cell microbes	Larvae	Stimulates worker feeding (nursing), inhibits foraging	Microbial communities on brood or in food may modify pheromone precursors or stability.	Hygienic behavior removes infected brood, altering microbial landscape and pheromone perception [14].
Foraging & Recruitment Signals	Nasonov gland pheromone (geraniol, citral, etc.)	Gut microbiome-influenced terpenoid metabolism	Workers	Orientation, swarm clustering, recruitment to resources	Microbial metabolism of dietary phytochemicals may provide precursors for terpenoid synthesis.	Pesticides impair microbial metabolism, potentially reducing precursor availability [17].
Alarm Pheromones	Isopentyl acetate (IPA)	Not directly microbially produced, but host synthesis may be metabolically supported	Workers	Defense, alarm recruitment	General host energy and acetyl-CoA metabolism, supported by microbial SCFAs, is required for IPA biosynthesis.	Energy crisis from dysbiosis (SCFA depletion) could limit the capacity to produce alarm signals (Figure 1).
Hive Atmosphere & Orientation Cues	Hive-specific volatile organic compound (VOC) blends	1. Gut microbiota (host-derived VOCs) 2. Hive microbes (in stored pollen, bee bread, honey)	Colony-wide	Hive identity, orientation, social homeostasis	Complex blend arises from host metabolism (influenced by gut microbes) and fermentation products of hive microbes (yeasts, bacteria).	Habitat change & antibiotics reduce microbial diversity in hive, simplifying VOC blend, potentially disorienting foragers [80,96]
Trophallaxis & Social Feeding Signals	Post-ingestive metabolites in nectar/honey	Gut microbiota of the donor bee	Workers	Nutrient sharing, information transfer, social immunity	Donor’s gut microbes metabolize food, altering its chemical profile before trophallaxis, potentially conveying health status.	Antibiotic exposure creates a dysbiotic “signature” that may be socially transmitted via trophallaxis [23,39]
Egg-marking Pheromones	Surface chemicals on eggs	Potential contribution of reproductive tract or ovipositor microbiota	Queen, Workers	Deterrence of worker egg-laying (queen’s eggs)	Microbes associated with the queen’s reproductive system may contribute to egg-surface chemistry, signaling egg identity.	Pathogens (e.g., Nosema) compromising queen health may alter associated microbiomes and egg signals.

Overall, honey bees are very extraordinary creatures, both at the individual bee level and as a superorganism, with the colony serving as the “individual.” Despite all of the stressors that bees endure, such as diseases, parasites, pesticides, and management, honey bees are resilient and possess a variety of features that combat these near-constant challenges [97]. Therefore, more research is required to understand how bees in colony conditions cope with these stresses, either alone or in combination, using various omics and metabolomics methodologies. Such knowledge is critical for sustainable beekeeping.

## 6. Translational Applications and a Roadmap for Precision Apiculture

This section distinguishes between translational applications that are currently supported by experimental or pilot-scale evidence and those that remain prospective, outlining near- and longer-term opportunities for validation and implementation.

### 6.1. Harnessing the Axis for Colony-Level Diagnostics and Interventions

Current/demonstrated: A mechanistic synthesis of the microbiome–metabolome axis has already produced testable biomarkers and pilot diagnostics in experimental and field settings. Non- or minimally invasive matrices—for example, hive-air volatilome, pooled hive debris, and bee bread—have been used in pilot and proof-of-concept studies to produce integrated signatures of colony physiological state. Portable membrane-inlet mass spectrometry (MIMS) and volatilomics workflows have been trialed in the field for rapid hive atmosphere profiling and pollutant/contaminant detection [98]. Volatile organic compound (VOC) profiling has been used to identify signatures associated with American foulbrood and other hive pathologies, providing a noninvasive early-warning approach that has been validated in controlled experiments and exploratory field studies [99]. Metabolomics of bee tissues and hemolymph has produced candidate biomarkers that track pesticide exposure and nutritional stress, and metabolomic biomarker-discovery work has established workflows and candidate panels for further validation [100]. Winter hive debris correlations with colony outcomes have also been reported, supporting the utility of pooled debris as an integrative, colony-level sample [101].

Near-term prospects/developmental: Translating these pilot results into routine management requires (a) analytical standardization (sample collection, storage, QC), (b) independent multi-site validation of candidate biomarkers, and (c) development of affordable, field-adapted detection platforms and decision-support tools. Once panels are validated, targeted metrics (for example, GSH/GSSG redox ratios, branched-chain amino-acid signatures, or SCFA ratios) could be implemented as early-warning indicators to trigger specific management responses [14].

Translational interventions—current evidence vs. prospects: Experimental trials have tested probiotics and postbiotics in lab and some field settings with mixed results: some controlled experiments report reductions in pathogen load or physiological benefits (for example, targeted lactobacilli formulations in controlled trials), while larger field trials of non-native, commercial probiotics have often failed to deliver colony-level benefits, underscoring the need for strain selection and context-specific validation [102,103]. Postbiotic or metabolite supplements (for example, microbially derived short-chain fatty acids or fermented pollen products) have shown physiological benefits in controlled work and therefore represent plausible postbiotic interventions to be piloted at the colony scale [15,104]. Functional restoration strategies, therefore, range from short-term (nutrient or postbiotic supplementation, already tested experimentally) to longer-term (engineered, strain-specific consortia designed to re-establish metabolic cross-feeding networks), which remain developmental and require regulatory and field efficacy studies.

Evaluation of these interventions should therefore combine individual-level endpoints (survival, pathogen load) and emergent colony-level metrics (foraging efficiency, hive weight dynamics, overwinter survival, and pollination performance). Where pilot diagnostics are already available, they should be used as outcome measures in randomized field trials to quantify effect sizes and guide regulatory adoption.

Practical examples (evidence from the literature guiding apicultural practices):

- Non-invasive VOC diagnostics for American foulbrood: VOC profiling identified a set of volatile biomarkers (e.g., 2,5-dimethylpyrazine and others) that distinguished *Paenibacillus larvae*-infected brood and were detectable in hive air—a concrete proof-of-concept for sensor development and early-warning detection. This work provides a direct path toward sensor prototypes for on-apiary screening [99].
- Portable MIMS for in-field hive atmosphere screening: a portable membrane-inlet mass spectrometer has been demonstrated for rapid detection of hive VOCs and even residues (e.g., pesticides) in situ, showing the feasibility of near-real-time field monitoring. This demonstrates how lab workflows can be miniaturized and brought to the apiary [98].
- Winter debris molecular surveillance: winter hive debris qPCR workflows have been shown to detect pathogen loads and several viral and bacterial agents noninvasively, supporting debris sampling as an operational surveillance matrix that can inform spring management decisions [101].
- Experimental probiotic interventions: defined lactobacilli-based “BioPatty” supplementation reduced *P. larvae* burdens in controlled and proof-of-concept experiments, showing that targeted probiotic delivery can influence larval disease dynamics under controlled conditions [103].
- Large-scale field tests of commercial probiotics: a longitudinal field study of commercial colonies found that widely marketed, non-native probiotic products did not rescue antibiotic-induced dysbiosis and were generally not beneficial under commercial management, demonstrating the gap between small experimental trials and large operational deployments and the need for rigorous field validation [102].

### 6.2. Pillars of a Precision Apiculture Framework

Current foundation (what exists now): A practical precision apiculture framework can be built around three pillars already supported by pilot work: (1) diagnostics (pilot VOC/mass-spec and metabolomic pipelines for hive-level sampling), (2) decision support (prototype data-integration and risk-scoring concepts), and (3) targeted mitigation (nutritional supplements and experimental probiotic/postbiotic approaches). For diagnostics, hive debris and bee bread are validated as integrative matrices in observational and exploratory studies and have been used to derive candidate metabolite and microbiome indicators [100,101]. Portable and field-adapted volatilomics platforms (e.g., MIMS) demonstrate the feasibility of near-real-time hive atmosphere screening [98].

Near-term operationalization (recommended, evidence-informed)—Diagnostics: Candidate biomarker panels (e.g., butyrate:acetate ratios, GSH/GSSG redox balance, amino-acid signatures) should be validated across diverse climates and management systems; targeted microbial functional assays (qPCR of butyrate-synthesis or xenobiotic-degradation genes) can contextualize metabolite shifts [14,105].

Decision support: Integrate biomarker outputs with environmental metadata (spray calendars, forage maps, weather, hive weight) to generate risk categories that drive specific, context-sensitive actions. Prototype decision-support systems exist conceptually and in pilot data-integration efforts, but they need field validation.

Targeted mitigation: Where diagnostic evidence points to specific deficits (e.g., depleted SCFAs, oxidative stress), employ targeted nutrition (glutathione precursors, phytochemical timing) or validated postbiotic supplements as first-line interventions; reserve community-scale microbial engineering for settings with demonstrated benefit in controlled field trials. Evidence on probiotic efficacy is mixed and often strain- and context-dependent; therefore, large, well-designed randomized field trials are required before broad adoption [102,103,104].

Longer-term vision (clearly prospective): Next-generation interventions (strain-specific, metabolically characterized consortia; regulatory-approved postbiotic products deployed at landscape scale) remain a promising but forward-looking goal that will require robust multi-site efficacy trials, safety assessment and cost–benefit analyses before routine implementation.

In short, pilot diagnostics and targeted nutritional/postbiotic interventions exist today as experimental tools and have informed small-scale management decisions in research and demonstration settings; larger-scale precision apiculture that relies on validated microbial consortia and automated, regulatory-accepted biomarker panels remains a near-term translational objective that will succeed only after multi-site validation and operational standardization [14,15].

## 7. Conclusions and Future Directions

The microbiome–metabolome axis is a sensitive interface through which diverse environmental and anthropogenic stressors can alter honey bee physiology, immunity, behavior and, ultimately, colony performance. Evidence synthesized here indicates recurrent convergence of chemically and biologically distinct stressors on shared microbial and metabolic pathways—changes in energy metabolism, shifts in short-chain fatty acids and amino-acid profiles, compromised antioxidant and detoxification capacity, and altered immune signaling. Importantly, multi-omics studies increasingly show that substantial functional disturbance can occur even when taxonomic shifts are modest, highlighting the value of functional readouts over taxonomic description alone.

At the same time, important limitations constrain confident inference and application. Many published studies are cross-sectional, small in sample size, or restricted to laboratory conditions; metabolite annotation and comparability across platforms remain incomplete; and integration across omics layers suffers from technical heterogeneity (batch effects, diverse extraction and analysis protocols) and limited temporal resolution. These factors complicate causal inference, increase the risk of confounding (for example, nutritional or pathogen covariates), and limit translation from controlled experiments to complex field settings. For these reasons, claims that link microbiome shifts directly to colony-level outcomes should be made cautiously and explicitly qualified unless supported by manipulative, field-validated evidence.

To advance mechanistic understanding and translational impact, we recommend the following experimental priorities.

1. Longitudinal, multi-site cohort studies. Establish temporally resolved sampling (seasonal to multi-year) across contrasting landscapes to capture natural variation, resilience and recovery trajectories and to distinguish transient from persistent alterations.
2. Controlled manipulations with field realism. Combine gnotobiotic and manipulative treatments (microbiome depletion/reconstitution, targeted probiotics, microbiome transplants) with semi-field/field deployments (mesocosms, apiary trials) to test causality under ecologically relevant conditions.
3. Multifactorial stressor experiments. Design factorial studies that jointly vary nutrition, pesticides, pathogens and temperature to reflect realistic exposure scenarios and to reveal interaction effects.
4. Standardization and benchmarking. Develop and adopt community standards for sampling, metabolite extraction, mass-spectrometry and sequencing pipelines, and for reporting metadata to enable cross-study synthesis and meta-analysis.
5. Functional validation of candidate metabolites and pathways. Prioritize biochemical and physiological assays (isotope tracing, enzyme assays, mitochondrial function tests, receptor assays) to move from correlation to mechanism for metabolites implicated by discovery omics.
6. Strain-resolved genomics and microbial culturing. Increase strain-level resolution and functional annotation of core taxa, expand culture collections, and link strain variation to metabolic phenotypes.
7. Integrative analytic frameworks and predictive biomarkers. Invest in causal inference tools (time-series modelling, perturbation experiments, validated machine-learning pipelines) and in development/validation of metabolome- or microbiome-derived biomarkers that are robust across sites and seasons.
8. Interdisciplinary coordination and data sharing. Encourage coordinated networks that pair omics with behavioral ecology, toxicology and regulatory science, and commit to open, well-annotated data deposition to accelerate replication and synthesis.

Collectively, these priorities will improve causal resolution, increase ecological realism, and accelerate translation of omics findings into robust biomarkers and management tools. Progress will require coordinated investment in field capacity, standardization, and cross-disciplinary training, but it is essential if we are to move from associative descriptions toward predictive, intervention-ready understanding of how the microbiome–metabolome axis shapes pollinator resilience.

## Data Availability

No new data were created or analyzed in this study. Data sharing is not applicable to this article.

## Supplementary Materials

The following supporting information can be downloaded at https://www.mdpi.com/article/10.3390/insects17030336/s1: Table S1. Summary of effects of major environmental and anthropogenic stressors on the gut microbiota–metabolome–host axis in honey bees. References [106,107] have been cited in supplementary file.
