# Correction: Al Naggar et al. How Environmental and Ecological Stressors Reprogram Honey Bee Chemistry Through the Microbiome–Metabolome Axis. *Insects* 2026, *17*, 336

**DOI:** 10.3390/insects17101003

**Published:** 2026-09-29

**Authors:** Yahya Al Naggar, Hamed A. Ghramh, Amira Elfarnawany, Amr Mohamed

**Affiliations:** 1 Central Labs, King Khalid University and Center of Bee Research and its Products (CBRP), King Khalid University, AlQura’a, P.O. Box 960, Abha 61413, Saudi Arabia; halgramh@kku.edu.sa; 2 Zoology Department, Faculty of Science, Tanta University, Tanta 31527, Egypt; amira.elfarnawany@science.tanta.edu.eg; 3 Biology Department, Faculty of Science, King Khalid University, P.O. Box 9004, Abha 61413, Saudi Arabia; 4 Department of Entomology, Faculty of Science, Cairo University, Giza 12613, Egypt; mamr@sci.cu.edu.eg

## Error in Affiliations

In the published publication [1], there was an error regarding the affiliations for Yahya Al Naggar. The correction is to merge the current affiliations 1 and 2 into a single affiliation, while keeping the current affiliation 3 unchanged as the second affiliation. The updated affiliations should therefore be:

1 Central Labs, King Khalid University and Center of Bee Research and its Products (CBRP), King Khalid University, AlQura’a, P.O. Box 960, Abha 61413, Saudi Arabia.
2 Zoology Department, Faculty of Science, Tanta University, Tanta 31527, Egypt.

The authors state that the scientific conclusions are unaffected. This correction was approved by the Academic Editor. The original publication has also been updated.

## References

[1] Al Naggar Y., Ghramh H.A., Elfarnawany A., Mohamed A. (2026). How Environmental and Ecological Stressors Reprogram Honey Bee Chemistry Through the Microbiome–Metabolome Axis. Insects.

