# Supplementary material for: How Environmental and Ecological Stressors Reprogram Honey Bee Chemistry Through the Microbiome–Metabolome Axis
Source: Insects. 2026 Mar 19;17(3):336. doi: 10.3390/insects17030336 (PMC13027192; doi:10.3390/insects17030336)
Supplement: Supplementary file 1 [file insects-17-00336-s001.zip › insects-4167146-supplementary.pdf]

Review Article

How Environmental and Ecological Stressors Reprogram Honey Bee Chemistry through the Microbiome–Metabolome Axis

Yahya Al Naggar [1,2,3,*], Hamed A. Ghramh [1,2,4], Amira Elfarnawany [3] and Amr Mohamed [5]

[1]Central Labs, King Khalid University, AlQura'a, P.O. Box 960, Abha 61413, Saudi Arabia; halgramh@kku.edu.sa

[2]Center of Bee Research and its Products (CBRP), King Khalid University, P.O. Box 960, Abha 61413, Saudi Arabia

[3]Zoology Department, Faculty of Science, Tanta University, Tanta 31527, Egypt; amira.elfarnawany@science.tanta.edu.eg

[4]Biology Department, Faculty of Science, King Khalid University, P.O. Box 9004, Abha 61413, Saudi Arabia

[5]Department of Entomology, Faculty of Science, Cairo University, Giza12613, Egypt; mamr@sci.cu.edu.eg

*Corresponding
Emil: yehia.elnagar@science.tanta.edu.eg

Table S1. Summary of effects of major environmental and anthropogenic stressors on the gut microbiota–metabolome–host axis in honey bees

Stressor	Gut Microbiota Effects	Metabolome Effects	Host Effects	Key References
**Pesticides**	Loss of core symbionts (*Snodgrassella*, *Gilliamella*, *Lactobacillus*); rise of opportunistic pathogens; microbial network instability	Disrupted carbohydrate, amino acid, lipid, and energy metabolism; reduced SCFAs; impaired detoxification; oxidative stress	Immunosuppression, reduced detoxification capacity, and increased disease susceptibility	[1–6]
**Antibiotics**	Long-term depletion of beneficial bacteria; incomplete recovery; socially transmitted dysbiosis	Reduced metabolic gene repertoire; impaired energy, amino acid, vitamin, and lipid metabolism	Reduced survival, delayed behavior, impaired immunity, and development	[7–11]
**Pathogens**	Shift toward opportunistic taxa; loss of protective symbionts; age- and season-dependent dysbiosis	Depletion of host energy metabolites, reduced antioxidant metabolites, and disease-specific metabolic signatures	Midgut damage, reduced lifespan, increased secondary infections	[12–16]
**Nutritional stress**	Reduced fermentative taxa; increased opportunists; enhanced *Nosema* establishment	Lower fermentative enzyme activity; impaired SCFA and carbohydrate metabolism	Reduced immunity, altered endocrine signaling, impaired development	[17–21]
**Heat stress**	Enrichment of heat-tolerant taxa; loss of protective symbionts under combined stress	Altered organic acid production; temperature-dependent metabolic shifts	Increased infection vulnerability; microbiome instability	[22–26]

Stressor	Gut Microbiota Effects	Metabolome Effects	Host Effects	Key References
**Habitat change**	Reduced microbial evenness; loss of beneficial taxa; enrichment of anthropogenic indicators	Reduced synthesis of essential metabolites (e.g., pyrimidines, PPP intermediates)	Reduced colony health, metabolic impairment	[27–31]
**Environmental contaminants**	Reduced alpha diversity; altered bacterial and fungal structure; loss of *Lactobacillus*	Enhanced detoxification, proteolysis, lipolysis; oxidative stress markers	Immune suppression, oxidative stress, impaired detoxification	[32–35]
**Shared effects across all stressors**	**Dysbiosis, reduced microbial stability and functional redundancy, and increased opportunistic pathogens**	**Disrupted energy metabolism, reduced SCFAs, impaired detoxification, and oxidative stress**	**Immune dysregulation, reduced resilience, higher susceptibility to disease, and colony decline**	

References

1. Di Prisco, G.; Cavaliere, V.; Annoscia, D.; Varricchio, P.; Caprio, E.; Nazzi, F.; Gargiulo, G.; Pennacchio, F. Neonicotinoid Clothianidin Adversely Affects Insect Immunity and Promotes Replication of a Viral Pathogen in Honey Bees. *Proc. Natl. Acad. Sci.* **2013**, *110* (46), 18466–18471. https://doi.org/10.1073/pnas.1314923110.

2. Kakumanu, M. L.; Reeves, A. M.; Anderson, T. D.; Rodrigues, R. R.; Williams, M. A. Honey Bee Gut Microbiome Is Altered by In-Hive Pesticide Exposures. *Front. Microbiol.* **2016**, *7*. https://doi.org/10.3389/fmicb.2016.01255.

3. Chen, H.; Wang, K.; Ji, W.; Xu, H.; Liu, Y.; Wang, S.; Wang, Z.; Gao, F.; Lin, Z.; Ji, T. Metabolomic Analysis of Honey Bees (Apis Mellifera) Response to Carbendazim Based on UPLC-MS. *Pestic. Biochem. Physiol.* **2021**, *179*, 104975. https://doi.org/10.1016/j.pestbp.2021.104975.

4. Shi, T.; Burton, S.; Wang, Y.; Xu, S.; Zhang, W.; Yu, L. Metabolomic Analysis of Honey Bee, Apis Mellifera L. Response to Thiacloprid. *Pestic. Biochem. Physiol.* **2018**, *152*, 17–23. https://doi.org/10.1016/j.pestbp.2018.08.003.

5. Qi, S.; Al Naggar, Y.; Li, J.; Liu, Z.; Xue, X.; Wu, L.; El-Seedi, H. R.; Wang, K. Acaricide Flumethrin-Induced Sublethal Risks in Honeybees Are Associated with Gut Symbiotic Bacterium Gilliamella Apicola through Microbe-Host Metabolic Interactions. *Chemosphere* **2022**, *307*, 136030. https://doi.org/10.1016/j.chemosphere.2022.136030.

6. Al Naggar, Y.; Singavarapu, B.; Paxton, R. J.; Wubet, T. Bees under Interactive Stressors: The Novel Insecticides Flupyradifurone and Sulfoxaflor along with the Fungicide Azoxystrobin Disrupt the Gut Microbiota of Honey Bees and Increase Opportunistic Bacterial Pathogens. *Sci. Total Environ.* **2022**, *849*, 157941. https://doi.org/10.1016/j.scitotenv.2022.157941.

7. Raymann, K.; Shaffer, Z.; Moran, N. A. Antibiotic Exposure Perturbs the Gut Microbiota and Elevates Mortality in Honeybees. *PLOS Biol.* **2017**, *15* (3), e2001861. https://doi.org/10.1371/journal.pbio.2001861.

8. Raymann, K.; Moran, N. A. The Role of the Gut Microbiome in Health and Disease of Adult Honey Bee Workers. *Current Opinion in Insect Science*. Elsevier Inc. April 1, 2018, pp 97–104. https://doi.org/10.1016/j.cois.2018.02.012.

9. Jia, S.; Wu, Y.; Chen, G.; Wang, S.; Hu, F.; Zheng, H. The Pass-on Effect of Tetracycline-Induced Honey Bee (Apis Mellifera) Gut Community Dysbiosis. *Front. Microbiol.* **2022**, *12*. https://doi.org/10.3389/fmicb.2021.781746.

10. Zhang, Z.; Mu, X.; Cao, Q.; Zhai, Y.; Zheng, L.; Liu, Y.; Zheng, H.; Zhang, X. Antibiotic Exposure Alters the Honeybee Gut Microbiota and May Interfere with the Honeybee Behavioral Caste Transition. *Insect Sci.* **2025**, *32* (1), 260–276. https://doi.org/10.1111/1744-7917.13374.

11. Shi, J.; Zhang, Y.; Liu, J.; Xu, M.; Wu, X. Larval Antibiotic Exposure Causes Persistent Impacts on Honeybees across Life Stages via Metabolic Succession. *J. Hazard. Mater.* **2025**, *497*, 139628. https://doi.org/10.1016/j.jhazmat.2025.139628.

12. Motta, E. V. S.; Moran, N. A. The Honeybee Microbiota and Its Impact on Health and Disease. *Nat. Rev. Microbiol.* **2024**, *22* (3), 122–137. https://doi.org/10.1038/s41579-023-00990-3.

13. Sbaghdi, T.; Garneau, J. R.; Yersin, S.; Chaucheyras-Durand, F.; Bocquet, M.; Moné, A.; El Alaoui, H.; Bulet, P.; Blot, N.; Delbac, F. The Response of the Honey Bee Gut Microbiota to Nosema Ceranae Is Modulated by the Probiotic Pediococcus Acidilactici and the Neonicotinoid Thiamethoxam. *Microorganisms* **2024**, *12* (1), 192. https://doi.org/10.3390/microorganisms12010192.

14. Dussaubat, C.; Brunet, J.-L.; Higes, M.; Colbourne, J. K.; Lopez, J.; Choi, J.-H.; Martín-Hernández, R.; Botías, C.; Cousin, M.; McDonnell, C.; Bonnet, M.; Belzunces, L. P.; Moritz, R. F. A.; Le Conte, Y.; Alaux, C. Gut Pathology and Responses to the Microsporidium Nosema Ceranae in the Honey Bee Apis Mellifera. *PLoS One* **2012**, *7* (5), e37017. https://doi.org/10.1371/journal.pone.0037017.

15. Li, Z.; Hou, M.; Qiu, Y.; Zhao, B.; Nie, H.; Su, S. Changes in Antioxidant Enzymes Activity and Metabolomic Profiles in the Guts of Honey Bee (Apis Mellifera) Larvae Infected with Ascosphaera Apis. *Insects* **2020**, *11* (7), 419. https://doi.org/10.3390/insects11070419.

16. Dosch, C.; Manigk, A.; Streicher, T.; Tehel, A.; Paxton, R. J.; Tragust, S. The Gut Microbiota Can Provide Viral Tolerance in the Honey Bee. *Microorganisms* **2021**, *9* (4), 871. https://doi.org/10.3390/microorganisms9040871.

17. Maes, P. W.; Rodrigues, P. A. P.; Oliver, R.; Mott, B. M.; Anderson, K. E. Diet-related Gut Bacterial Dysbiosis Correlates with Impaired Development, Increased Mortality and Nosema Disease in the Honeybee (Apis Mellifera). *Mol. Ecol.* **2016**, *25* (21), 5439–5450. https://doi.org/10.1111/mec.13862.

18. Ricigliano, V. A.; Anderson, K. E. Probing the Honey Bee Diet-Microbiota-Host Axis Using Pollen Restriction and Organic Acid Feeding. *Insects* **2020**, *11* (5), 291. https://doi.org/10.3390/insects11050291.

19. Branchiccela, B.; Castelli, L.; Corona, M.; Díaz-Cetti, S.; Invernizzi, C.; Martínez de la Escalera, G.; Mendoza, Y.; Santos, E.; Silva, C.; Zunino, P.; Antúnez, K. Impact of Nutritional Stress on the Honeybee Colony Health. *Sci. Rep.* **2019**, *9* (1), 10156. https://doi.org/10.1038/s41598-019-46453-9.

20. Powell, J. E.; Lau, P.; Rangel, J.; Arnott, R.; De Jong, T.; Moran, N. A. The Microbiome and Gene Expression of Honey Bee Workers Are Affected by a Diet Containing Pollen Substitutes. *PLoS One* **2023**, *18* (5), e0286070. https://doi.org/10.1371/journal.pone.0286070.

21. Castelli, L.; Branchiccela, B.; Garrido, M.; Invernizzi, C.; Porrini, M.; Romero, H.; Santos, E.; Zunino, P.; Antúnez, K. Impact of Nutritional Stress on Honeybee Gut Microbiota, Immunity, and Nosema Ceranae Infection. *Microb. Ecol.* **2020**, *80* (4), 908–919.

22. Moghadam, N. N.; Thorshauge, P. M.; Kristensen, T. N.; de Jonge, N.; Bahrndorff, S.; Kjeldal, H.; Nielsen, J. L. Strong Responses of Drosophila Melanogaster Microbiota to Developmental Temperature. *Fly (Austin).* **2018**, *12* (1), 1–12. https://doi.org/10.1080/19336934.2017.1394558.

23. Palmer-Young, E. C.; Raffel, T. R.; McFrederick, Q. S. Temperature-Mediated Inhibition of a Bumblebee Parasite by an Intestinal Symbiont. *Proc. R. Soc. B Biol. Sci.* **2018**, *285* (1890), 20182041. https://doi.org/10.1098/rspb.2018.2041.

24. Palmer-Young, E. C.; Ngor, L.; Burciaga Nevarez, R.; Rothman, J. A.; Raffel, T. R.; McFrederick, Q. S. Temperature Dependence of Parasitic Infection and Gut Bacterial Communities in Bumble Bees. *Environ. Microbiol.* **2019**, *21* (12), 4706–4723. https://doi.org/10.1111/1462-2920.14805.

25. Palmer-Young, E. C.; Markowitz, L. M.; Huang, W.-F.; Evans, J. D. High Temperatures Augment Inhibition of Parasites by a Honey Bee Gut Symbiont. *Appl. Environ. Microbiol.* **2023**, *89* (10). https://doi.org/10.1128/aem.01023-23.

26. Van Wyk, J. I.; Beirne, L.; Bowder, S.; Campbell, E.; Disharoon, M.; Dreyer, M.; Frolichstein-Appel, N.; Gill, A.; Jones-Ducharme, A.; Kinkaid, A.; Korr, G. B.; McCabe, M.; McDowell, E.; Perez, F.; Villarreal-Mentz, R.; Johnston, J. Hot and Bothered, Bees' Gut Microbiome Shifts Under Thermal Stress and Pathogen Infection. November 7, 2025. https://doi.org/10.1101/2025.11.06.687019.

27. Gorrochategui-Ortega, J.; Muñoz-Colmenero, M.; Kovačić, M.; Filipi, J.; Puškadija, Z.; Kezić, N.; Parejo, M.; Büchler, R.; Estonba, A.; Zarraonaindia, I. Publisher Correction: A Short Exposure to a Semi-Natural Habitat Alleviates the Honey Bee Hive Microbial Imbalance Caused by Agricultural Stress. *Sci. Rep.* **2022**, *12* (1), 22237. https://doi.org/10.1038/s41598-022-25981-x.

28. Drew, G. C.; Budge, G. E.; Frost, C. L.; Neumann, P.; Siozios, S.; Yañez, O.; Hurst, G. D. D. Transitions in Symbiosis: Evidence for Environmental Acquisition and Social Transmission within a Clade of Heritable Symbionts. *ISME J.* **2021**, *15* (10), 2956–2968. https://doi.org/10.1038/s41396-021-00977-z.

29. Budge, G. E.; Adams, I.; Thwaites, R.; Pietravalle, S.; Drew, G. C.; Hurst, G. D. D.; Tomkies, V.; Boonham, N.; Brown, M. Identifying Bacterial Predictors of Honey Bee

Health. *J. Invertebr. Pathol.* **2016**, *141*, 41–44. https://doi.org/10.1016/j.jip.2016.11.003.

30. Nguyen, P. N.; Rehan, S. M. Wild Bee and Pollen Microbiomes across an Urban–Rural Divide. *FEMS Microbiol. Ecol.* **2023**, *99* (12). https://doi.org/10.1093/femsec/fiad158.

31. Potts, S. G.; Biesmeijer, J. C.; Kremen, C.; Neumann, P.; Schweiger, O.; Kunin, W. E. Global Pollinator Declines: Trends, Impacts and Drivers. *Trends Ecol. Evol.* **2010**, *25* (6), 345–353. https://doi.org/10.1016/j.tree.2010.01.007.

32. Rothman, J. A.; Leger, L.; Kirkwood, J. S.; McFrederick, Q. S. Cadmium and Selenate Exposure Affects the Honey Bee Microbiome and Metabolome, and Bee-Associated Bacteria Show Potential for Bioaccumulation. *Appl. Environ. Microbiol.* **2019**, *85* (21). https://doi.org/10.1128/AEM.01411-19.

33. Wang, K.; Li, J.; Zhao, L.; Mu, X.; Wang, C.; Wang, M.; Xue, X.; Qi, S.; Wu, L. Gut Microbiota Protects Honey Bees (Apis Mellifera L.) against Polystyrene Microplastics Exposure Risks. *J. Hazard. Mater.* **2021**, *402*, 123828. https://doi.org/10.1016/j.jhazmat.2020.123828.

34. Li, Z.; Guo, D.; Wang, C.; Chi, X.; Liu, Z.; Wang, Y.; Wang, H.; Guo, X.; Wang, N.; Xu, B.; Gao, Z. Toxic Effects of the Heavy Metal Cd on Apis Cerana Cerana (Hymenoptera: Apidae): Oxidative Stress, Immune Disorders and Disturbance of Gut Microbiota. *Sci. Total Environ.* **2024**, *912*, 169318. https://doi.org/10.1016/j.scitotenv.2023.169318.

35. An, T.; Feng, W.; Li, H.; Wu, Y.; Dai, P.; Liu, Y.-J. Combined Effects of Microplastics and Flupyradifurone on Gut Microbiota and Oxidative Status of Honeybees (Apis Mellifera L.). *Environ. Res.* **2025**, *270*, 121026. https://doi.org/10.1016/j.envres.2025.121026.
